# A spatial analysis of lime resources and their potential for improving soil magnesium concentrations and pH in grassland areas of England and Wales

**DOI:** 10.1038/s41598-021-98735-w

**Published:** 2021-10-14

**Authors:** T. Bide, E. L. Ander, M. R. Broadley

**Affiliations:** 1 British Geological Survey, Keyworth, Nottingham, NG12 5GG UK; 2 School of Biosciences, University of Nottingham, Sutton Bonington Campus, Nottinghamshire, LE12 5RD UK

**Keywords:** Geochemistry, Agroecology

## Abstract

Magnesium (Mg) is essential for animal health. Low Mg status (hypomagnesaemia) can be potentially fatal in ruminants, like cattle and sheep, and is widespread in Europe with economic impacts on farming. The application of Mg-rich agricultural lime products can help to ensure pasture forage consumed by animals contains sufficient Mg and, in areas of low pH, has the dual benefit of reducing soil acidity to levels best suited for grass production. This aim of this study was to determine if Mg-rich lime products could be used in a more effective manner in agricultural production systems. Potential resources of carbonate rocks (limestone, dolostone and chalk) in the UK, and their Mg:Ca status were identified, using datasets from the British Geological Survey (BGS). These data were combined with the locations of agricultural lime quarries, and areas where soils are likely to be deficient in Mg and/or require liming. Areas of potential demand for Mg-rich agricultural lime include areas in south east Wales, the Midlands and North East England. Although, areas where this may be an effective solution to low soil Mg values are restricted by the availability of suitable products. Conversely, areas of low soil pH in England and Wales are often found close to quarries with the ability to supply high Ca limes, suggesting that the low rates of lime use and liming is not due to supply factors. This study provides information that can help to guide on-farm decision making for use of Mg-rich and other lime resources. This could be used in conjunction with other options to reduce risks of Mg deficiency in livestock, and improve soil pH.

## Introduction

### Magnesium soil contents and relationships to livestock health

Magnesium (Mg) deficiency (hypomagnesaemia) in ruminant livestock is a serious issue for the agricultural sector and accounts for a significant number of animal deaths annually. It is caused by a diet deficient in Mg, or due to an imbalance in the supply of Mg in comparison to other mineral cations^1^. Hypomagnesaemia is likely to be responsible for lower-productivity and diminished well-being in more animals in a herd compared to those displaying acute symptoms, given that herds/flocks generally receive a common diet^2,3^. If Mg deficiency could be prevented, it would be of benefit to both animal welfare and economic productivity. Recent research has confirmed that whilst hypomagnesaemia is commonly reported by UK farmers, the reported use of preventative measures is low, and the use of pasture interventions is lower still^4^. Pasture interventions can include the application of Mg-rich fertiliser or lime products, or selection of sward species with a propensity to take-up elevated Mg concentrations^4,5^.

One aspect of dietary supply is the geographic control of pasture and farm-produced fodder. It is known that total Mg and plant-available Mg concentrations in soil are controlled by geological and geographic factors^6–9^ and that there is little evidence for any changes in pasture soil Mg concentrations through time^6,10^. The magnesium content of soil relates to that of the bedrock, where it is high in the bedrock it is high in soil and vice versa. Thus, the composition of all pasture and farm-grown fodder will be influenced by this natural environmental endowment as well as pasture management decisions.

### Grass productivity and soil pH

A key pasture management activity is that of soil pH—which here is reported as measured in water (pH_w_), consistent with standard agronomic laboratory practice in the UK^11^. Grassland mineral soil is recommended to be maintained at pH_w_ ≥ 6.0 in Britain^12,13^. In Ireland, where grass-clover pasture is more widely practiced, a pH_w_ threshold of 6.5 is recommended^14^. However, multiple lines of evidence exist that indicate pasture soil is frequently below these pH_w_ recommendations. Private sector on-farm sample data summaries from the UK consistently show pH typically below recommendations in pasture soil: the most recent annual data synthesis reports 57% of grassland soil with pH_w_ ≤ 5.99, and 27% with pH_w_ 6.00–6.49^8^. This is consistent with systematically collected public sector data across the north of Ireland, where 84% of pasture samples were below the clover-grass recommended threshold of pH_w_ 6.5^15^.

**Figure 1:**
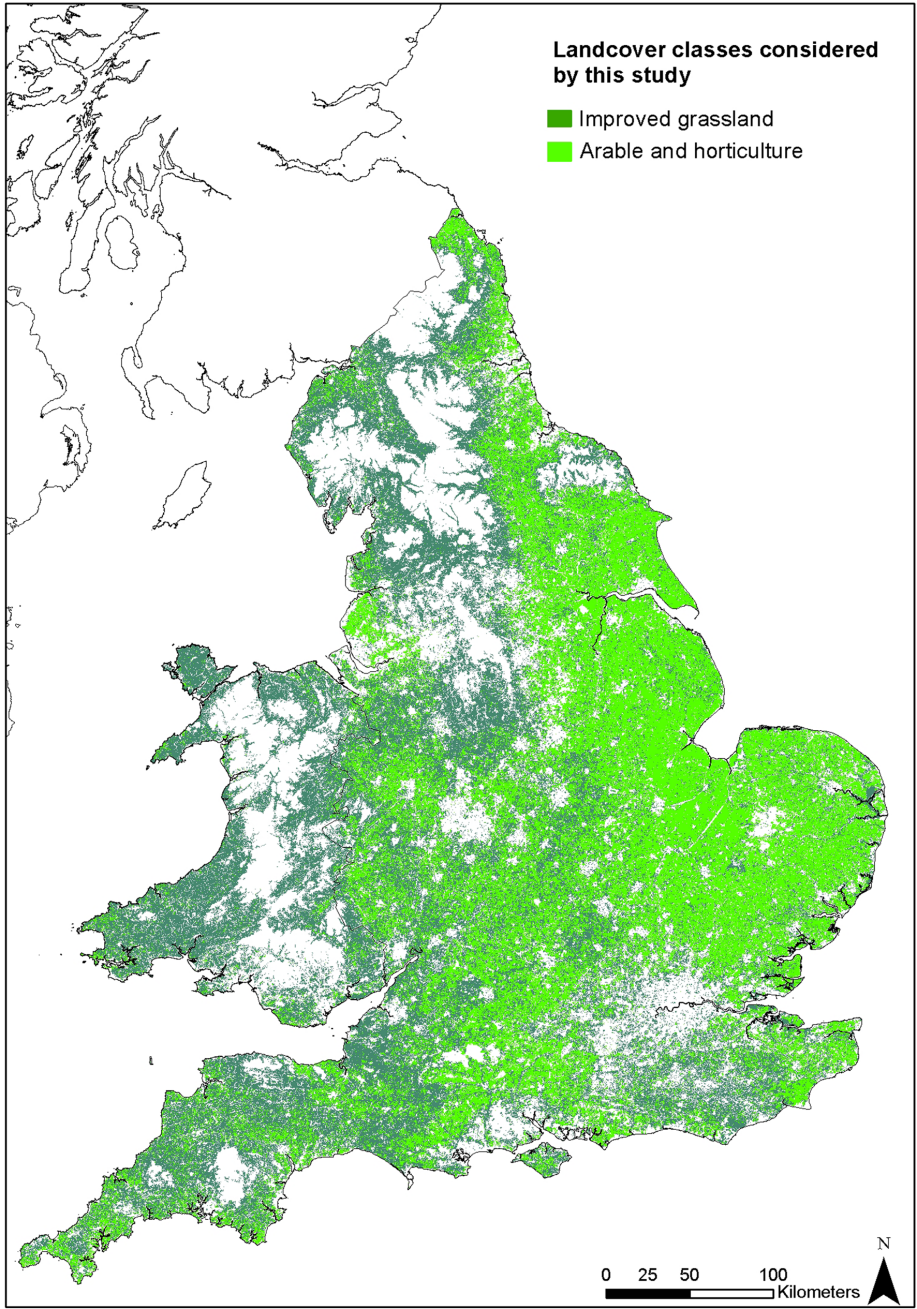
Classes of land cover considered by this study, based on Land Cover Map 2015^33^. Some features of this map are based on digital spatial data licensed from the UK Centre for Ecology and Hydrology. Created using ArcMap 10.7.1, ESRI, 2019.

**Figure 2:**
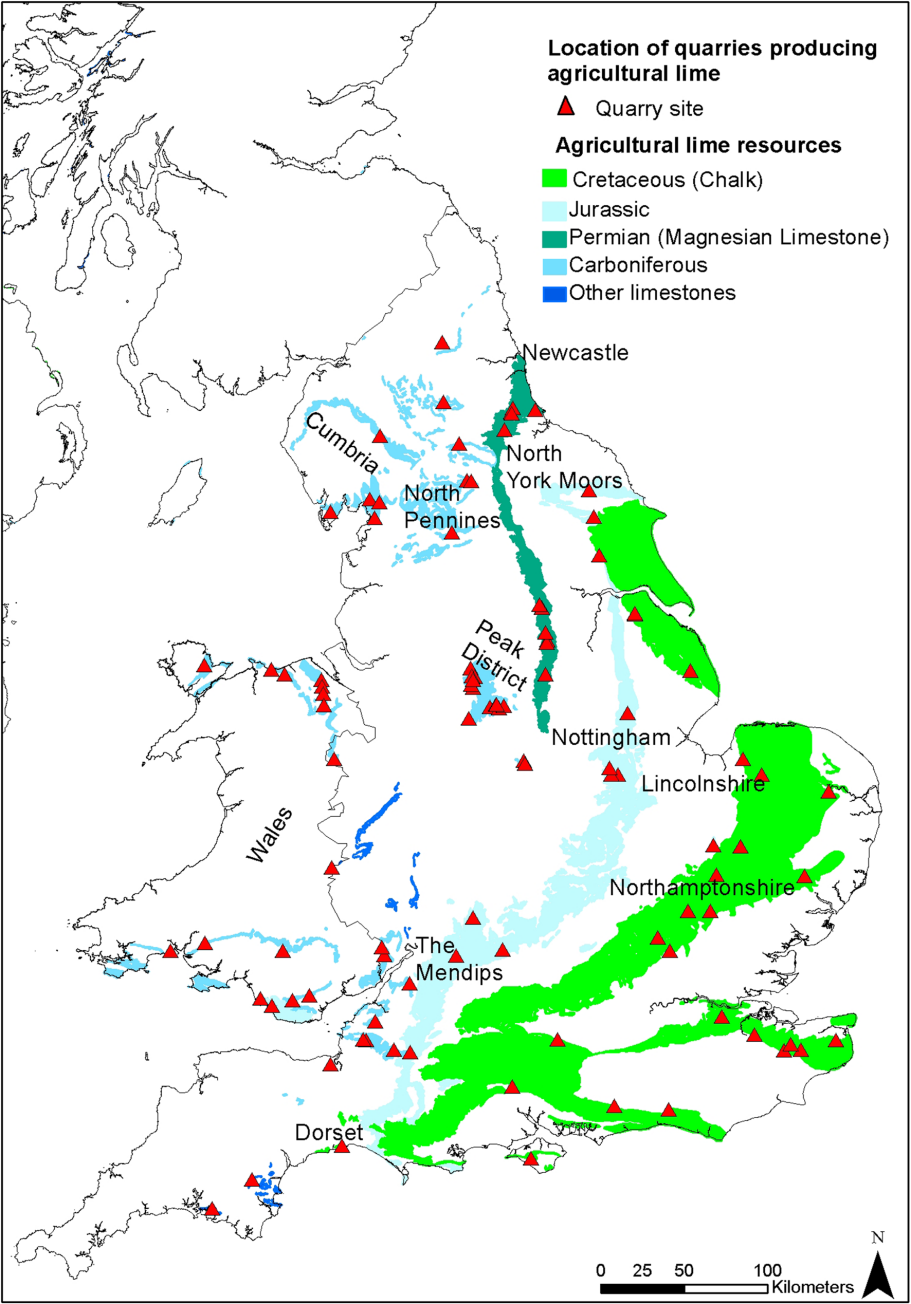
The outcrop of the major carbonate rocks of England and Wales and the location of agricultural lime quarries. Adapted from BGS Geology 50K^34^. Created using ArcMap 10.7.1, ESRI, 2019.

Grassland production is widespread in Wales and western England (Fig. 1). Two environmental factors jointly contributing to the lower pH in these regions are (1) geological—these areas are most often on soils which are developed over rocks with low concentrations of base cations (Figs. 1 and 2); and, (2) these areas are also often upland areas, associated with typically higher rainfall^16^ which will further leach base cations. Added to these environmental factors are the application of nitrogen fertilisers which have an acidifying effect^17^. Thus, many pastoral areas require treatment using agricultural lime in order to optimize soil pH for grass growth^18^.

### The use of liming materials

The opportunity to improve grazing livestock Mg nutrition through use of Mg-rich lime is identified in guidance available to farmers^12^. This can have the dual benefits of maintaining soil pH for grass growth and ensuring Mg levels in livestock feed is at sufficient levels^19–21^. The combination of soil treatment for pH and Mg would therefore appear to be an efficient solution to solve issues surrounding Mg deficiency^22^. Conversely, for many soils with existing high Mg levels it may be important to treat with low Mg liming materials to ensure an optimal Ca–Mg balance to preserve the soil structure^23^.

The use of Mg lime is only one of many methods of controlling Mg levels in livestock feed. Other methods, such as direct additions to feeds, salt licks, pelletised fertilisers products are also effective in reducing incidences of hypomagnesaemia and need to be considered as part of holistic review of a individual farms requirements, this is discussed in Kumssa et al.^5^.

Maintaining optimal soil pH will directly affect the productivity of grass used for grazing, and will increase fertiliser use efficiency^14^. However, in some cases, for example upland sheep farming, a low investment—low return approach, with minimum interventions such as liming, may be entirety sensible and appropriate to the farm business and local landscape^24,25^.

The extent to which agricultural lime is used in Britain is captured through the annual British Survey of Fertiliser Practice (BSFP) and can also be inferred from commodity production statistics. Production can be regarded as a good proxy for consumption since due to its high bulk and low price it is not exported in significant quantities.

**Figure 3:**
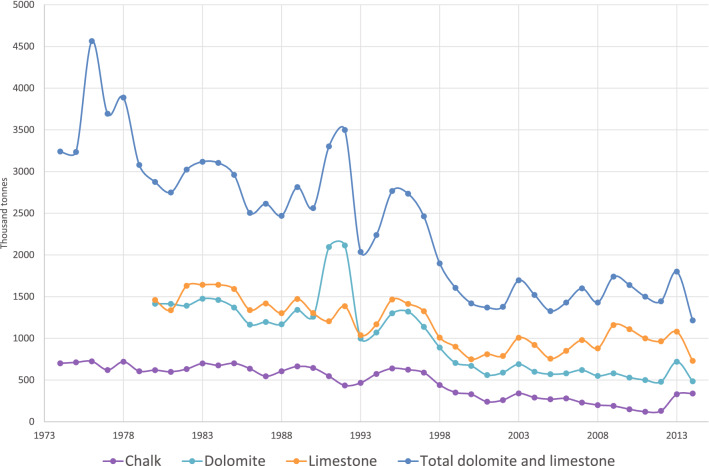
Great Britain production of chalk, dolomite and limestone for agricultural use source:^32^.

The BSFP, is an annual Department for Environment, Food and Rural Affairs (DEFRA) survey^26^, which representatively samples fertiliser and lime use across the British farming sector. This captures information on lime use in three geological material categories as used in arable and pastoral systems, as well as use of sugar beet lime and ‘other’ options. Sugar beet lime use is very low on grassland (generally unrecorded on ‘permanent’ pasture); ‘other’ categories are generally on a par with Mg-lime, but more detailed liming characteristics are not reported. Figure 3 shows a clear trend in decreasing production of agricultural liming material over the last 40 years. Lime use in the UK peaked in the late 1950s and mid 1960s likely due to a subsidy for agricultural lime in place at the time, this ended in 1978, causing prices to increase and subsequently lime use to decrease^27^. The use of agricultural lime has continued on a declining, or flat trend, likely due to reluctance to engage in soil treatments that are seen to be costly and a lack of knowledge over its potential benefits.

**Figure 4:**
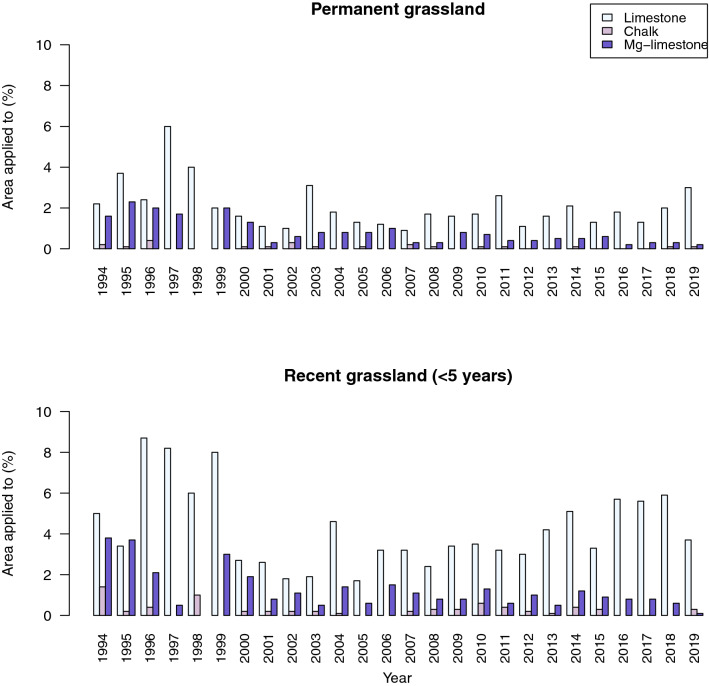
Limestone, chalk and high magnesium limestone (Mg-lime) application to (**a**) permanent and (**b**) recent grassland for Great Britain, from the annual British Survey of Fertiliser Practice 1994–2019 publications DEFRA^26^, https://www.gov.uk/government/collections/fertiliser-usage). Note that data for limestone and chalk are combined in 1997 reporting; data were reported to one decimal place for data other than 1998–1999, which were reported as integers.

Figure 4 shows the use of geological lime products to be low at present in respect of the proportion of fields to which lime is reported to be applied, and that this is particularly pervasive on permanent grassland, with a 10-year average to 2019 of 2.9% (range 2.0–4.1%). Of this, a 10-year average of 0.4% of fields had Mg-lime applied, with 1.8% of fields having limestone applied. The limited use of chalk (0.1% of fields) probably reflects the distance between the majority of pasture and the outcrop of the chalk. Recent grassland (< 5 years) has a 10-year average of 6.2% of fields, with an average of 0.8% of fields having Mg-lime applied (Fig. 4).

The low use of lime on farms and the low current supply of agricultural lime suggest that currently pastoral land is being under-limed, and this is entirely consistent with the generally low pH of pasture soil reported^8,15^. Although this relationship is complicated by factors such as the removal of the lime subsidy in 1970s.

### Geological resources for agricultural lime in England and Wales

Agricultural lime is any calcium (Ca) or magnesium (Mg) carbonate rich form of crushed rock (limestone, dolostone or chalk) that is applied to soil in order that the high Ca, or Ca + Mg, concentration raises and neutralises the pH of the soil. Individual lime products have different neutralising values, for example, burnt lime which is ground to a fine powder will release CaO into the soil much more rapidly than coarse crushed limestone rock due to its increased solubility. However, the vast majority of agricultural lime in the UK consists of ground limestone or chalk due to the higher bulk and considerably lower prices of these materials when compared to processed products. UK limestones are principally valued for their use as construction aggregates (around 70% of material quarried is for this purpose)^28^. Agricultural lime has a much smaller market share, less than 5% of the total sales of limestone^28^.

Carbonate rocks are distributed in discrete areas across England and Wales (Fig. 2), accounting for ~ 13% of the total area; whilst all alkaline in reaction, these rocks have a wide variety of physical properties and variation in their chemistry, which can affect their efficacy when used as agricultural lime. These properties are intrinsically linked to the sedimentary environments in which these rocks were deposited as well as how they have been altered by post-depositional processes. As such, they need to be treated separately when considering their use as agricultural limestone, which is thus mandated in international agricultural lime product standards^29^.

Carbonate rocks that constitute raw materials for agricultural lime comprise limestone and dolostone. Limestone is a sedimentary rock composed mainly of calcium carbonate (CaCO_3_) whereas dolostone is composed mainly of magnesium carbonate in the form of the mineral dolomite (CaMg(CO_3_)_2_). These carbonate rocks can contain variable proportions of other carbonate minerals and non-carbonate impurities, mainly in the form of siliciclastic, clay and other accessory minerals^30^. British limestones can be broadly differentiated in their physical–chemical properties according to the geological era in which they were deposited.

In terms of geographic extent the Cretaceous aged chalk of south eastern and eastern England covers the largest area, followed by Carboniferous limestones and dolostones of the Mendips, North and South Wales, Peak District, North Pennines and Cumbria. Permian aged limestone (which predominately comprises dolomite) is also an important carbonate rock, outcropping between Newcastle and Nottingham. Jurassic limestones also occur extensively across Dorset, Northamptonshire, Lincolnshire and the North York Moors. Older limestones of Ordovician, Silurian and Devonian ages are also present across England and Wales to lesser extents. The broad outcrop of these limestones is shown on Fig. 2.

The Cretaceous Chalk is typically softer than other limestones, with lower levels of non-carbonate minerals and thus high Ca contents: this can be advantageous for liming as it is easy to work, crush and will effectively neutralise acidic soils. Jurassic limestones typically are heterogeneous with high levels of impurities, and can be associated with layers of mudrocks and siltstones, but they can also be thickly bedded and relatively soft. Permian limestones, like Jurassic limestones, are known for being soft and lithologically heterogeneous, but they are principally comprised of dolomite. The Carboniferous Limestone is formed of much more competent (stronger) rocks and these are therefore valued principally for their aggregate properties. They can be chemically very pure, or associated with interbedded muds and silts, and they can also be variably and locally dolomitised. Older Ordovician, Silurian and Devonian limestones occur in much smaller areas and have variable properties but have proved to be important resources on a local scale and are predominantly utilised for aggregate applications rather than agricultural lime^9,31^.

### Study aim

This study aims to develop simple spatial approaches, for England and Wales, to aid in the decision making process around the application of agricultural limes. Specifically, whether soil pH improvement by liming may be a cost effective solution and whether it may be beneficial to lime using high Mg materials for both pH benefits and to protect livestock against hypomagnesaemia. These approaches are fully documented to ensure they can be replicated elsewhere.

## Methodology

This study aims to assess the potential for the supply and demand for agricultural lime in general, and Mg-rich agricultural lime in particular, through the analysis of existing data.

To identify the relevant datasets the analysis was split into two aspects; supply and demand. The supply side data focuses on the current state of the industry and the location of resources. Whereas the demand side looks at geographic areas of potential need for liming materials.

### Supply data

Data for the location of sites producing agricultural lime was compiled using the BritPits database, a comprehensive database of mines and quarries in the UK^35^. This is updated on an annual basis licensed by the British Geological Survey but active sites are available to viewer via the GeoIndex data viewer (https://mapapps2.bgs.ac.uk/geoindex/home.html). This relational database was queried using search terms for 'Active Sites' and the following end uses: 'agricultural industry end use'; 'lime' and 'agricultural lime'.

Compositional data for lime products is not in the public domain, and generally considered confidential by the quarrying industry. Chemical data for lime products was therefore extracted from legacy data reports and geological mapping data. It was assumed that the specification of material sold would be the same as the composition of the surface bedrock geology which was being extracted using mapped information at 1:50,000 scale for England and Wales^36^. This dataset, however, does not separate carbonate rocks suitable for agricultural lime from carbonate rocks that are used for aggregate or other industrial purposes. Thus the data were modified, based on a geological review, to best represent the spatial distribution of resources suitable for agricultural lime as an end use. The geological information was therefore attributed with chemical data to understand their potential as high Mg or Ca feedstock materials using Harrison et al.^9^ ‘Appraisal of high-purity limestones in England and Wales, part 1 resources’ which reports the average Ca and Mg values of carbonate rocks in England and Wales. This publication is focused on high specification industrial uses of limestone, but the results are also applicable to agricultural lime applications. A significant caveat with using this approach is that these sedimentary rocks can be complex and very heterogeneous. Small changes in the depositional environment during formation of the deposit can lead to large variations in rock type and the physical and chemical properties such as its neutralising value or Ca:Mg ratio, which is important when considering high Mg limes. Therefore, the chemical values used for this study represent an average for a particular formation and could vary substantially locally. All chemical values were categorised according to EU guidance of the specifications required for different grades of material^29^.

The geological review shows that simple rules could be applied for many geological formations to categorise according to the chemical composition. Permian dolomitised limestones have some of the highest Mg values of all carbonate rocks in the study, commonly exceeding 20%. Dolostone formations from the Carboniferous Limestone have similarly high Mg contents. Both these groups could be directly attributed using BGS 1:50,000 geological mapping^34^. More problematic are limestones that are either very variable in nature (often with high siliciclastic sediment contents) or have been variably dolomitised. These units include the heterogeneous Permian aged Cadeby and Brotherton Formations which occur in a north–south band across central and northern England as well as variably dolomitised Carboniferous limestones in south-western and north-western England, as defined by BGS geological mapping data^34^. Generally, most other carbonate rocks, including all Cretaceous Chalk, have low-Mg values (< 2%) and primarily comprise calcium carbonate.

**Figure 5:**
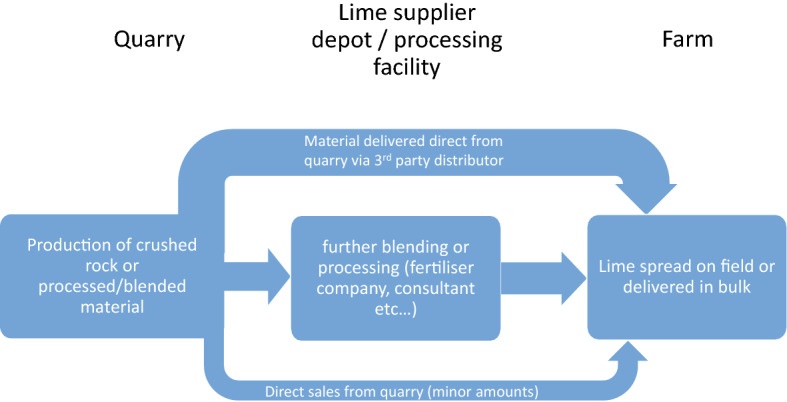
Major flows of agricultural lime in the UK.

Due to its low cost per tonne, transport can form a substantial part of overall costs, potentially limiting how far material is transported. No data is available for agricultural lime transport distances. Typical and maximum distances to market for aggregate was used as a proxy because they are a similar low price—high volume material. Whilst imperfect it would appear the prices for both can be similar taking price statistics for aggregates^32^ and price per tonne is the most significant factor in determining transport distances. However, in reality, the supply chain will be considerably more complex, because value can be added for lime through contracts for both soil pH testing and lime spreading, as well as supply. Transportation can also be affected by factors such as utilising return loads from other bulk haulage. A broad outline of the flows of agricultural lime from quarry to farm are outlined in Fig. 5.

The most comprehensive industry consultation to date reported 60 km as the maximum transportable distance by road for aggregates^37^. This will almost certainly now be higher, as can be seen by rising average transport distances for aggregates, where smaller operations have been closed and mothballed as the industry has consolidated^38^, and the same change is also true of the agricultural lime industry (Agricultural Lime Association, personal communication, September 2019). Therefore, the average distance of 45 km^38^ was taken, and a maximum transport distance of 80 km was applied in this study.

The road network for the study area was taken from the freely available official dataset OS OpenRoads^39^. This dataset only includes A and B roads and motorways which was judged sufficient resolution for this study and the fact that heavy goods vehicles avoid the use of minor roads. Different road types were not, however, differentiated in the analysis as no cost data was available for travel on different road types.

### Demand data

Information on the demand for agricultural lime in different areas of the UK requires a spatial understanding of where grasslands may have pH values that indicate liming would be beneficial, and where those may coincide with low plant-available soil Mg concentrations. If pasture or arable land requires liming, how much material may be required can be calculated using a relationship between soil texture, soil pH and land use. This relationship has been used to created calculators (Table 1) that will predict the amount of lime required^12,40,41^ which we use as the framework in this study.

**Table 1:** Liming requirements look-up table, adapted from Agriculture and Horticulture Development Board^12^. ^a^For mineral and organic soils, the target soil pH is 6.7 for continuous arable cropping and 6.2 for grass. Aim for 0.2 units above the optimum pH. ^b^For peaty soils, the target soil pH is 6.0 for continuous arable cropping and 5.5 for grass. Aim for 0.2 units above the optimum pH.

Soil type	Sands and loamy sands		Sandy loams and silt loams		Clay loams and clays		Organic soils_a_		Peaty soils_b_	
Original category from BGS Soil parent material model^45^	SAND + LOAM SAND > LOAM SAND > LOAM > CLAY SAND ALL (if the grain size was argillic—arenaceous)		LOAM > CLAY > SAND LOAM LOAM > SAND > CLAY LOAM > SAND		CLAY CLAY > LOAM CLAY + LOAM CLAY > SAND LOAM > CLAY		ALL (only if the grain size was peat)			
Land use	Arable	Grass	Arable	Grass	Arable	Grass	Arable	Grass	Arable	Grass
	t/ha of lime required									
Initial soil pH of 6.2	3	0	4	0	4	0	4	0	0	0
Initial soil pH of 6.0	4	0	5	0	6	0	6	0	0	0
Initial soil pH of 5.5	7	3	8	4	10	4	10	4	8	0
Initial soil pH of 5.0	10	5	12	6	14	7	14	7	16	6

**Figure 6:**
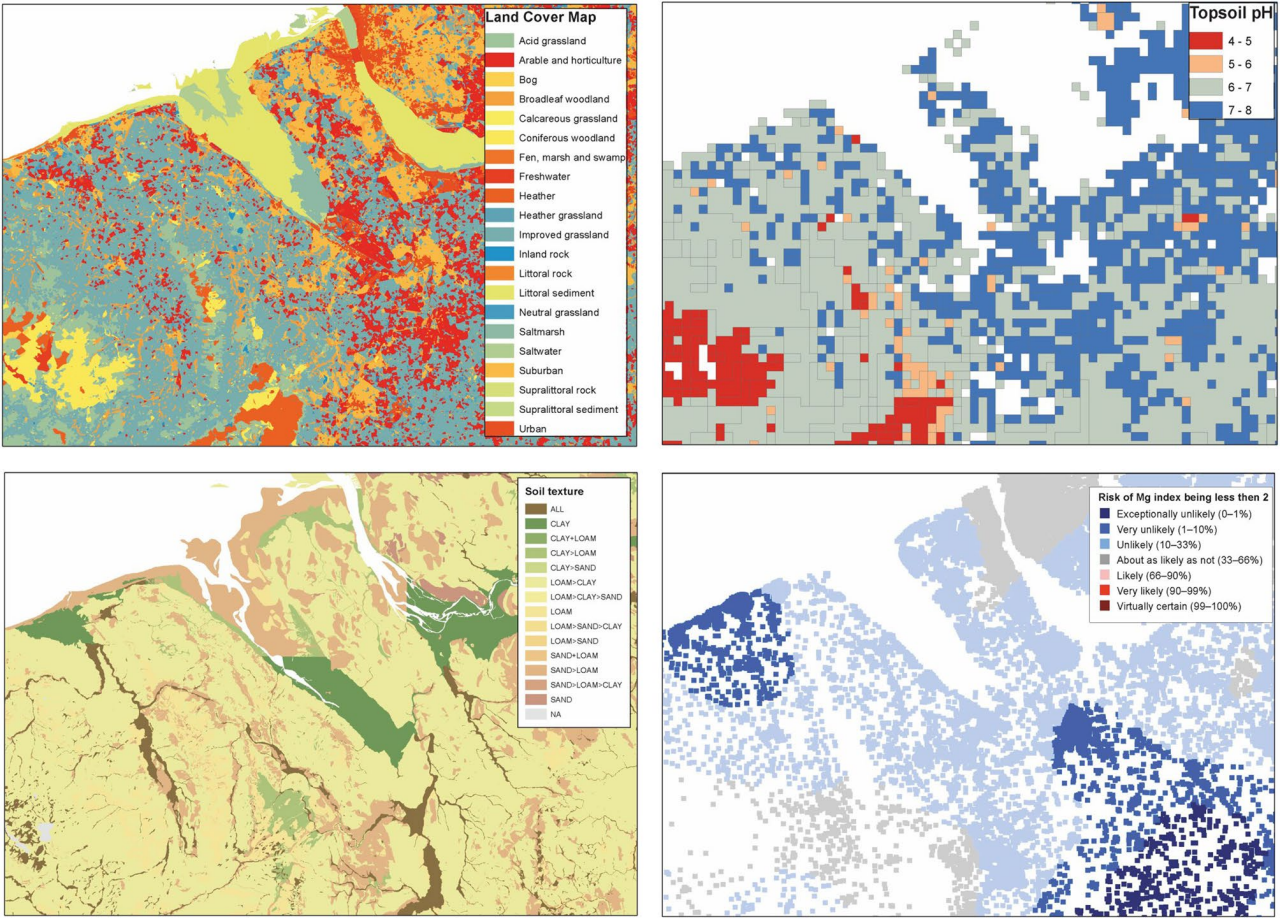
examples of input datasets to define areas of potential lime demand, all available on UKSO.org. Clockwise from top left: land use^33^, pH^43^, risk of Mg deficiency^6^, soil parent material Model soil texture^45^. The top two excerpts are based on digital spatial data licensed from the UK Centre for Ecology and Hydrology. Created using ArcMap 10.7.1, ESRI, 2019.

Areas of pasture were defined using the LandCover Map (Fig. 6), LCM2015 data^33^ downloaded from the UK Soil Observatory (UKSO) platform^42^, which records land use derived from spectral analysis of Landsat-7 imagery, reported by a 25 m × 25 m raster grid. Different categories of grassland are recorded: Acid grassland; Arable and horticulture; Calcareous grassland; Heather grassland; Improved grassland and Neutral grassland. Only improved grassland and arable and horticulture classes were selected for analysis here. In the case of other grassland categories, such as rough grasslands and upland grassland areas, it was considered unlikely that improvement via liming would be feasible or appropriate.

A key factor in determining whether applying agricultural lime would have a beneficial effect on crop yield is soil pH. We took the data for 1 km square pH estimates for Great Britain from the Centre for Ecology and Hydrology (CEH) dataset^43^ (Fig. 6). The estimates are based upon 3145 soil samples 0–15 cm depth with analysis in water as described by Avery and Bascomb^44^.

Soil texture affects how agricultural lime will break down and deliver calcium and magnesium to the soil. It is therefore is critical to understand whether liming may be required and if so what quantity of material is needed. Data for soil texture was derived from the BGS Soil Parent Material Model (SPMM) (Fig. 6), which was designed to facilitate spatial mapping of soil parameters^45^. The numerous categories for soil texture were condensed into the five categories which are defined by lime requirement calculators^12,40,41^ as shown in Table 1.

The ‘All’ category used in the soil parent material maps was problematic as this could encompass a variety of soil types, generally either peats or siliclastic sediments, these however could be differentiated using the grain size attribute in the dataset to ensure the correct soil texture was attributed. The data from the SPMM also made it impossible to differentiate between the two classes of organic soils defined by the lime requirement calculators. These were therefore combined into one class for organic soils and peat. Very few areas (less than 1%) of arable land or improved grassland correspond to areas of organic soils and peat as defined by the LCM2015 data and the SPMM, so these assumptions make little impact to the final dataset. After the mapping exercise was complete, a subset dataset was created using the classification system used by the lime requirement calculators.

Lark et al.^6^ produced spatial estimates of plant-available soil Mg and the risk of Mg deficiencies in soil based on geostatistical analysis of two soil geochemical datasets (Fig. 6). These data are available via UKSO. This dataset highlights the areas most at risk from Mg deficiency based on the Agriculture and Horticulture Development Board (AHDB) defined Mg soil indices^12^, which is the guidance followed by the agriculture sector across the area of this study. These areas of risk of Mg deficiency were used to define areas where Mg lime may be required for this study.

### Spatial data analysis

ArcGIS (ESRI) version 10.3.1 was used for all spatial analysis. The Network Analysis Tool was used to measure distances, from quarries used as input facilities in the analysis (or subsets of these based on predicted Mg or Ca contents of products), along the road network (OSOpenRoads) used as the Network Dataset. To calculate areas which are likely to have a demand for agricultural lime datasets for land cover, pH, Mg deficiency and soil texture were linked with the spatial join function, this functionality was also used to link quarry data to geochemical properties.

## Results

A critical component of this study was to identify areas of demand for liming materials and if these areas could also benefit from increased Mg soil concentrations. This was done by defining areas where the application of agricultural lime was predicted to have a beneficial impact on crop yields. This required continuous data for England and Wales for land use, soil pH, soil texture and soil Mg levels as described in the “Methodology” section. The primary component was the land use as without suitable land use for pasture no other factors were relevant. Around 115,000 km^2^ (74%) of the study area was classified as a land use with potential for pasture that may be suitable for liming, however, much of this will be arable land as opposed to land for grazing. Data for soil pH also played a critical role as no liming would be required if the soil pH was already at recommended levels. Data for pH did not cover the entire study area, some areas had no values attributed to them, these were, however, mainly urban areas so did not have a large impact on the spatial analysis (only 0.2% of land use suitable for liming had no pH data that could be associated with it). The CEH pH dataset showed soil pHs in land suitable for liming ranges from 6–8 with the majority around or just over 6. After integration of all datasets spatial analysis shows that 18% of the study area may be both suitable for and require liming with Ca liming material and 8% with Mg liming material.

The supply factors considered for agricultural lime included the location of production sites, composition of the products and market penetration. In total 96 potential supply sites were identified (these do not include lime produced from food manufacture by-products and also includes sites which may only supply lime for cement and mortar uses). Of these only 14 have the potential to produce high Mg lime and 29 have the potential to produce high Ca lime. These sites, like limestone resources, are widely spread across England and Wales. High Mg sites were restricted to the Permian Magnesian Limestone Formation due to pervasive dolomitisation which occurred in these rocks soon after formation, likely due to recirculation of fluids in a restricted basin environment^46^ and Carboniferous dolostones principally caused by post diagenetic alteration from hydrothermal fluids^47^. The geological history is relevant here as it indicates that the former will be almost entirely dolomitised (Mg-enriched) and the latter only partially dolomitised.

### Results of spatial analysis

**Figure 7:**
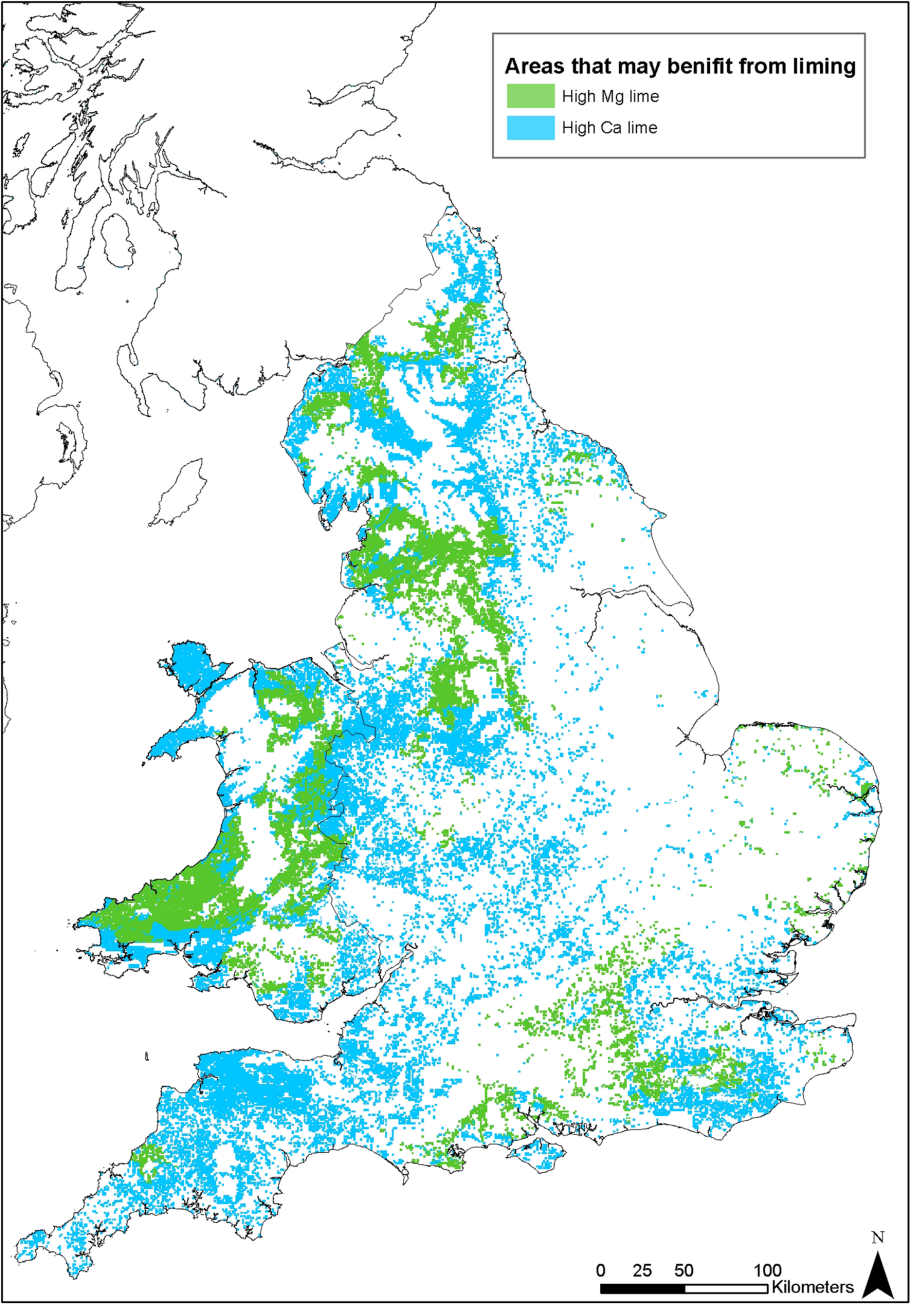
The results of the demand analysis showing areas where the application of agricultural limes may be beneficial. Some features of this map are based on digital spatial data licensed from the UK Centre for Ecology and Hydrology. Created using ArcMap 10.7.1, ESRI, 2019.

Figure 7 shows the results of the demand analysis, the areas of improved grassland that would benefit from liming (in light blue), and if an Mg lime would be beneficial (in green). This is a product of integrating soil texture, soil pH, land use, and soil Mg indices data.

**Figure 8:**
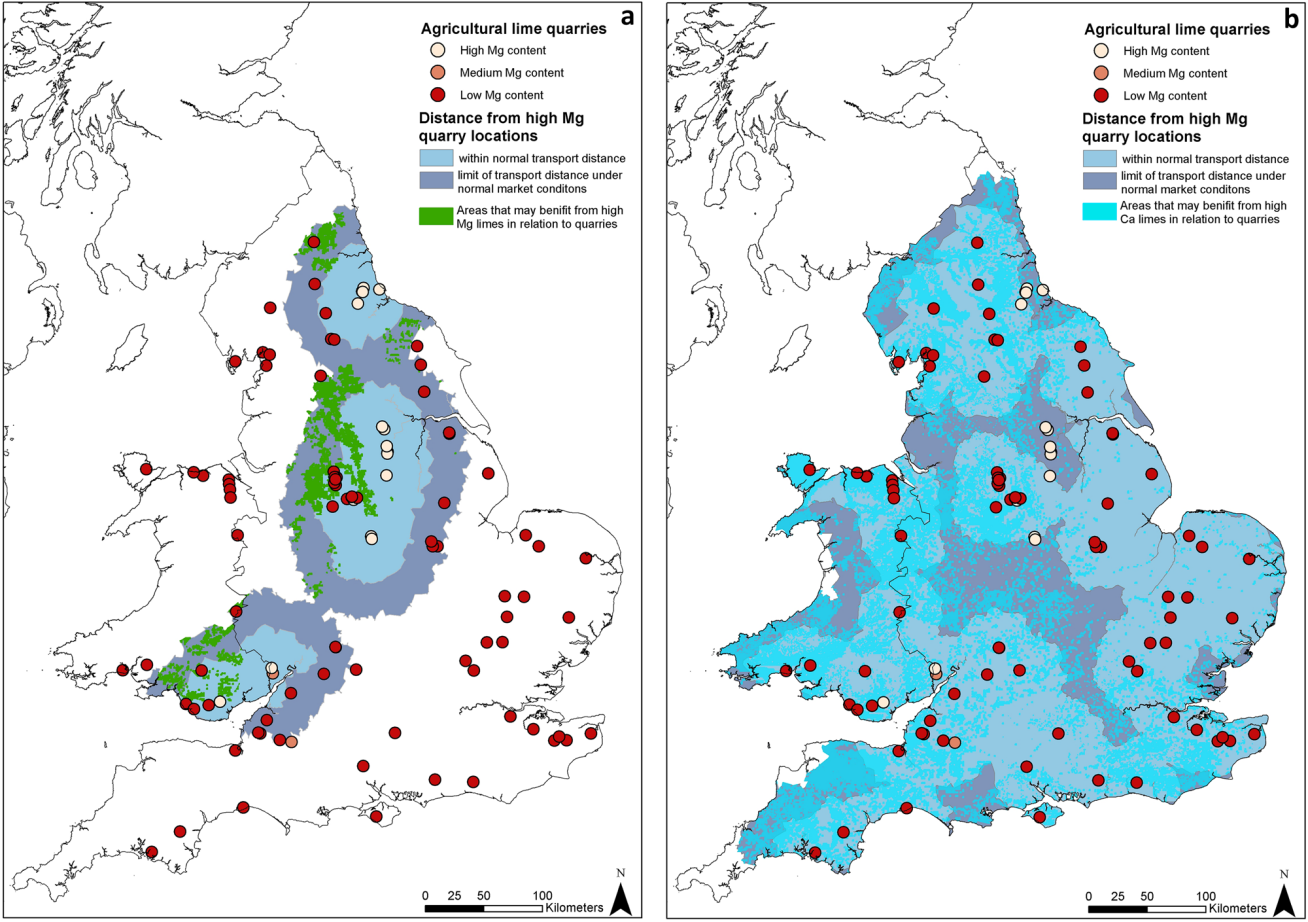
The results of the supply/demand analysis, (**a**) estimated normal and maximum transport distances from quarries supplying high Mg limes and the areas within them that may benefit from high Mg limes. (**b**) Estimated normal and maximum transport distances from quarries supplying low Mg (and therefore high Ca limes) and the areas within them that may benefit from high Ca limes. Created using ArcMap 10.7.1, ESRI, 2019.

The result of the network analysis using the supply and demand information is shown in Fig. 8. For high Mg quarries, when using the typical distances (45 km, blue) or typical maximum distance (80 km, grey) that high Mg lime may travel from source, the restricted area where this may be an effective and practical option for soil improvement can be seen. Only 15% of areas that may benefit from the application of high Mg limes are within the maximum transport distance from supply sites, decreasing to 6% using the typical transport distance. This is in contrast with high Ca lime, for which much of England and Wales is in areas in proximity to quarries that can supply this material. Jointly using both types of quarries (not shown) the vast majority of England and Wales are in the range of a source of agricultural lime: 74% of areas that require liming are within the maximum transport distance and 55% the typical transport distance. The exceptions are parts of mid Wales, south west and north east England.

## Discussion

This spatial analysis shows that for many areas the distance from source to market for Mg-rich liming material is substantial so other methods of Mg intervention need to be considered, for example by application of processed Mg additives or planting of Mg-rich forage^5^. This is the case for many areas that have Mg deficient soils, as highlighted by areas that may be benefited by the application of high Mg limes in Fig. 7, such as central and south west Wales, Devon, the south east of England, Cheshire, Lancashire, Cumbria and Northumberland. This is perhaps unsurprising as soil around areas where bedrock is enriched in Mg will likely itself not be at risk of having low Mg soils. This is coupled with the fact the distribution of high Mg carbonate rocks in England and Wales is restricted to relatively small areas. However, for some areas, such as around the East Midlands, South Yorkshire and south west Wales the application of high Mg limes could be a practical solution for livestock Mg deficiencies. For these broad areas spatial analysis, as shown by Figs. 7 and 8, show clear overlaps of areas of potential supply with potential demand. These areas are typically defined geologically as areas of sandstones (i.e. Coal Measures in the East Midlands and south east Wales), which generally have poor soil qualities due to the high silica contents of the bedrock soil parent materials. However, these areas are also in close proximity to high Mg limestones, either from dolomitised Carboniferous limestones (Wales) or from the outcrop of high Mg Permian limestones (Midlands).

This spatial analysis also shows areas where soil Mg vales are already high and, if liming were to be used to treat soil pH, the use of low Mg, high Ca material may be beneficial to avoid deleterious Mg levels, which can, in some circumstances, inhibit the uptake of other soil nutrients. For example around the outcrop of the Permian magnesian limestone in the Midlands and North East England. For some of these areas the nearest quarry supplying high Ca limes may be up to 80 km away, making sourcing limes of the appropriate chemistry a concern. There are very few areas in England or Wales that are outside the potential supply range of quarries that can supply high Ca liming materials. This is due to the widespread nature of limestones and a well-developed extractive industry, as shown by Fig. 8. This suggests that Ca rich agricultural limes should be readily available in the UK and that the reduction in the use of agricultural limes is not due to supply issues but other market factors.

This study only considers improved grassland and arable land with low pH. In many upland grass areas with acidic soils soil treatment is impractical, may cause harm to delicate grassland ecosystems and is uneconomic as a low maintenance/low yield approach is more appropriate^24,25^. These types of land cover may be protected, or may be valued for their specific biodiversity as a unique function of soil chemistry. For these reasons the inclusion of upland grass areas was not considered useful for an accurate representation of areas that may benefit from liming and justified specifically excluding them. Although in some cases the application of lime to upland areas can also have positive environmental impacts^48^ and these will need to be assessed on a case by case basis. The exclusion of upland grassland areas was a simple process as they are separately classed, "Heather grassland" and "Acid grassland", in the land use dataset used.

This study only deals with crushed and ground liming products that comprise untreated quarried and crushed geological material. There are also a wide range of other products that have been processed in some form, i.e. pelletised lime as well as products derived from non-geological materials, such as sugar beet waste. The market dynamics of these products are in no way comparable to ground lime due to higher costs, due to processing, although the overall effects of these products to soil parameters are broadly similar^19^. It is also important to consider negative environmental effects of the use of liming products. There is little data regarding different footprints of crushed rock agricultural lime vs more processed products, however lime quarries are generally small operations, with minimal processing requirements. The CO_2_ emissions of the use of agricultural lime are relatively small due to reactions during the breakdown of lime, which sequester CO_2_^49^.

Due to limited data availability, transport distance used in this study rely on many assumptions. In reality market factors may lead to a very variable end price where transport distances may exceed those of the raw material. How far the material will travel is dependent on a complex interplay of local markets (e.g. utilising return loads from other freight traffic) and the nature of contracts between end users and suppliers which may include testing and mitigation for a range other soil improvement measures. There are examples of Mg limes being transported, by road, several hundred miles from northern England to Scotland, however these are exceptional (Agricultural Lime Association, personal communication, September 2019). Although not factored into figures used in this study, due to lack of data, there is evidence that Mg limes will have greater transport distances when compared to low Mg alternatives due to its ability to increase soil Mg indices increasing its value (Agricultural Lime Association, personal communication, September 2019).

It is labour and resource intensive to identify and treat areas of low pH and Mg and so there would seem to be substantial benefits in tools for preliminary assessments of soil improvement interventions. The simple tools developed by this study show how integration of the many already available and easily accessible spatial datasets and legacy survey data can be developed into such decision making toolkits. Such integration of datasets can also add to the understanding of the supply and demand dynamics of agricultural lime in England and Wales, ensuring that the most realistic and appropriate resource management strategies are applied. It is hoped that provision of such toolkits can aid in ensuring that soil treatment techniques are used to their maximum potential for enhancing productivity.

## Conclusions

High Mg and Ca agricultural limes may have wider market use than is currently realised, as shown in farm-practice and trade statistics data. This analysis specifically shows that, in certain areas, the use of high Mg lime for soil treatment is a viable possibility for the duel benefit of altering pH and increasing soil Mg contents. Also shown is the wide distribution and existing supply chain for high Ca limes, these products are readily available for the vast majority for lowland grasslands in England and Wales. This approach also opens up the opportunity to compare the likely usefulness of liming as a tool to reduce the risks of hypomagnesaemia, in conjunction with the assessment of other approaches which can be used on-farm. Pasture management options should always be selected after analysis of soil and/or forage to ensure an appropriate course of action is taken given local conditions. This analysis shows how it is possible to create useful relevant new datasets that can benefit new users from the huge range of existing, publicly available, legacy datasets, that may not always appear accessible to potential users outside their immediate domain, e.g. geological sciences.

## References

[1] Schonewille JT (2013). Magnesium in dairy cow nutrition: An overview. Plant Soil.

[2] Robinson DL, Kappel LC, Boling JA (1989). Management practices to overcome the incidence of grass tetany. J. Anim. Sci..

[3] Foster A, Livesey C, Edwards G (2007). Magnesium disorders in ruminants. In Pract..

[4] Kumssa DB (2019). A reconnaissance survey of farmers’ awareness of hypomagnesaemic tetany in UK cattle and sheep farms. PLoS One..

[5] Kumssa DB (2020). Magnesium biofortification of Italian ryegrass (*Lolium*
*multiflorum* L.) via agronomy and breeding as a potential way to reduce grass tetany in grazing ruminants. Plant Soil.

[6] Lark R, Ander E, Broadley M (2019). Combining two national-scale datasets to map soil properties, the case of available magnesium in England and Wales. Eur. J. Soil Sci..

[7] McGrath SP, Loveland PJ (1992). The Soil Geochemical Atlas of England and Wales.

[8] Professional Agricultural Analysis Group. Collation of data from routine soil analysis in the UK (2017).

[9] Harrison, D., Hudson, J. & Cannell, B. Appraisal of high purity limestone in England and Wales. British Geological Survey technical report; WF/90/10. 1–18 (British Geological Survey Nottingham, 1994).

[10] Baxter SJ, Oliver MA, Archer JR (2006). The Representative Soil Sampling Scheme of England and Wales: The spatial variation of topsoil nutrient status and pH between 1971 and 2001. Soil Use Manag..

[11] van Reeuwijik LP (2002). Procedures for Soil Analysis.

[12] Agriculture and Horticulture Development Board (2019). Nutrient Management Guide (RB209), Section 1 Principles of nutrient management and fertiliser use.

[13] Farm Advisory Service. Fertiliser recommendations for grassland. Technical note TN726 (2019).

[14] Coulter BS, Lalor S (2008). Major and Micro Nutrient Advice for Productive Agricultural Crops.

[15] Lark M, Ander L, Knights K, Young M (2016). Unearthed: Impacts of the Tellus Surveys of the North of Ireland.

[16] Simpson I, Jones P (2012). Updated precipitation series for the UK derived from Met Office gridded data. Int. J. Climatol..

[17] Goulding KWT (2016). Soil acidification and the importance of liming agricultural soils with particular reference to the United Kingdom. Soil Use Manag..

[18] Johnston A, Whinham W (1980). The use of lime on agricultural soils. Proc. Fertil. Soc..

[19] Higgins S, Morrison S, Watson CJ (2012). Effect of annual applications of pelletized dolomitic lime on soil chemical properties and grass productivity. Soil Use Manag..

[20] Riggs KS, Syers JK, Rimmer DL, Sumner ME (1995). Effect of liming on calcium and magnesium concentrations in herbage. J. Sci. Food Agric..

[21] Jokinen R (1982). Effect of liming on the value of magnesium sulphate and two dolomitic limestones as magnesium sources for ryegrass. Agric. Food Sci..

[22] Pires AL, Ahlrichs JL, Rhykerd CL (1991). Hybrid ryegrass response to acid soil treatment with calcitic and dolomitic lime. Commun. Soil Sci. Plant Anal..

[23] Keren R (1991). Specific effect of magnesium on soil erosion and water infiltration. Soil Sci. Soc. Am..

[24] Dicks LV (2019). What agricultural practices are most likely to deliver “sustainable intensification” in the UK?. Food Energy Secur..

[25] Office for National Statistics. Fertiliser usage on farms: Results from the Farm Business Survey, England 2018/19 (2020).

[26] DEFRA (Department for Environment Food and Rural Affairs). British Survey of Fertiliser Practice. (HM Government, 2020).

[27] Holland J (2018). Liming impacts on soils, crops and biodiversity in the UK: A review. Sci. Total Environ..

[28] Idoine NE, Bide T, Brown TJ, Raycraft ER (2016). United Kingdom Minerals Yearbook 2015: Statistical data to 2014. 83.

[29] European Commission. COMMISSION REGULATION (EU) No 463/2013. *Off. J. Eur. Union* **304** (2013).

[30] Mitchell C (2011). High purity limestone quest. Ind. Minerals.

[31] Conybeare WD, Phillips W (2014). Outlines of the Geology of England and Wales.

[32] Ministry of Housing Communities and Local Government. Annual minerals raised inquiry survey (2015).

[33] Rowland, C. S. *et al.* Land Cover Map 2015 (vector, GB) (NERC Environmental Information Data Centre, https://catalogue.ceh.ac.uk/documents/6c6c9203-7333-4d96-88ab-78925e7a4e73, 2017).

[34] British Geological Survey. DiGMapGB 50 version 8 (http://digimap.edina.ac.uk, 2016).

[35] Cameron, D. User guide for the BRITPITS GIS dataset (2013).

[36] British Geological Survey. The mineral resources of the UK at 1:50 000 (British Geological Survey, 2012).

[37] Highley, D., Chapman, G. R. & Bonel, K. The economic importance of minerals to the UK. British Geological Survey report CR/04/070N (British Geological Survey, 2004).

[38] Mineral Products Association. *MPA sustainability portal: Transport* (https://mineralproducts.org/documents/MPA_SD_Report_2018.pdf, 2018).

[39] Ordnance Survey. OS Open Roads, November 2019 (https://www.ordnancesurvey.co.uk/business-government/products/open-map-roads, 2019).

[40] Tzilivakis J, Lewis K, Green A, Warner D (2002). ALA Lime Calculator.

[41] Goulding KWT, McGrath SP, Johnston AE (1989). Predicting the lime requirement of soils under permanent grassland and arable crops. Soil Use Manag..

[42] British Geological Survey. *UK Soil Observatory* (http://mapapps2.bgs.ac.uk/ukso/home.html, 2019).

[43] Henrys PA, Keith AM, Robinson DA, Emmett BA (2012). Model estimates of topsoil pH and bulk density [Countryside Survey].

[44] Avery BW, Bascomb CL (1974). Soil Survey Laboratory Methods.

[45] British Geological Survey. Soil Parent Material Model (British Geological Survey, https://www.bgs.ac.uk/products/onshore/soilPMM.html, 2014).

[46] Raymond LR (1962). The petrology of the lower magnesian limestone of north-east Yorkshire and south-east Durham. Q. J. Geol. Soc..

[47] Ford TD (2002). Dolomitization of the carboniferous limestone of the Peak District: A review. Mercian Geol..

[48] McCallum HM (2016). A role for liming as a conservation intervention? Earthworm abundance is associated with higher soil pH and foraging activity of a threatened shorebird in upland grasslands. Agric. Ecosyst. Environ..

[49] West TO, McBride AC (2005). The contribution of agricultural lime to carbon dioxide emissions in the United States: Dissolution, transport, and net emissions. Agric. Ecosyst. Environ..

